# Interim Estimates of 2016–17 Seasonal Influenza Vaccine Effectiveness — United States, February 2017

**DOI:** 10.15585/mmwr.mm6606a3

**Published:** 2017-02-17

**Authors:** Brendan Flannery, Jessie R. Chung, Swathi N. Thaker, Arnold S. Monto, Emily T. Martin, Edward A. Belongia, Huong Q. McLean, Manjusha Gaglani, Kempapura Murthy, Richard K. Zimmerman, Mary Patricia Nowalk, Michael L. Jackson, Lisa A. Jackson, Angie Foust, Wendy Sessions, LaShondra Berman, Sarah Spencer, Alicia M. Fry

**Affiliations:** ^1^Influenza Division, National Center for Immunization and Respiratory Diseases, CDC; ^2^University of Michigan, Ann Arbor, Michigan; ^3^Marshfield Clinic Research Foundation, Marshfield, Wisconsin; ^4^Baylor Scott and White Health, Texas A&M University Health Science Center College of Medicine, Temple, Texas; ^5^University of Pittsburgh Schools of the Health Sciences and University of Pittsburgh Medical Center, Pittsburgh, Pennsylvania; ^6^Group Health Research Institute, Seattle, Washington.

In the United States, annual vaccination against seasonal influenza is recommended for all persons aged ≥6 months (*1*). Each influenza season since 2004–05, CDC has estimated the effectiveness of seasonal influenza vaccine to prevent influenza-associated, medically attended, acute respiratory illness (ARI). This report uses data, as of February 4, 2017, from 3,144 children and adults enrolled in the U.S. Influenza Vaccine Effectiveness Network (U.S. Flu VE Network) during November 28, 2016–February 4, 2017, to estimate an interim adjusted effectiveness of seasonal influenza vaccine for preventing laboratory-confirmed influenza virus infection associated with medically attended ARI. During this period, overall vaccine effectiveness (VE) (adjusted for study site, age group, sex, race/ethnicity, self-rated general health, and days from illness onset to enrollment) against influenza A and influenza B virus infection associated with medically attended ARI was 48% (95% confidence interval [CI] = 37%–57%). Most influenza infections were caused by A (H3N2) viruses. VE was estimated to be 43% (CI = 29%–54%) against illness caused by influenza A (H3N2) virus and 73% (CI = 54%–84%) against influenza B virus. These interim VE estimates indicate that influenza vaccination reduced the risk for outpatient medical visits by almost half. Because influenza activity remains elevated (*2*), CDC and the Advisory Committee on Immunization Practices recommend that annual influenza vaccination efforts continue as long as influenza viruses are circulating (*1*). Vaccination with 2016–17 influenza vaccines will reduce the number of infections with most currently circulating influenza viruses. Persons aged ≥6 months who have not yet been vaccinated this season should be vaccinated as soon as possible.

Methods used by the U.S. Flu VE Network have been published previously (*3*). At five study sites, patients aged ≥6 months seeking outpatient medical care for an ARI with cough, within 7 days of illness onset, were enrolled.* Study enrollment began after ≥1 laboratory-confirmed cases of influenza were identified through local surveillance for ≥2 consecutive weeks. Patients were eligible for enrollment if they 1) were aged ≥6 months on September 1, 2016, and thus eligible for vaccination; 2) reported an ARI with cough and onset ≤7 days earlier; and 3) had not been treated with influenza antiviral medication (e.g., oseltamivir) during this illness. After obtaining informed consent from patients or parents/guardians for their children, participants or their proxies were interviewed to collect demographic data, general and current health status, symptoms, and 2016–17 influenza vaccination status. Respiratory specimens were collected from each patient using nasal and oropharyngeal swabs, which were placed together in a single cryovial with viral transport medium. Only nasal swabs were collected for patients aged <2 years. Specimens were tested at U.S. Flu VE Network laboratories using CDC’s real-time reverse transcription – polymerase chain reaction (rRT-PCR) protocol for detection and identification of influenza viruses. Participants (including children aged <9 years who require 2 vaccine doses during their first vaccination season) were considered vaccinated if they received ≥1 dose of any seasonal influenza vaccine ≥14 days before illness onset, according to medical records and registries (at Wisconsin site), medical records and self-report (at Texas and Washington sites), or self-report only (Michigan and Pennsylvania sites). VE was estimated as 100% x (1 - odds ratio).^†^ Estimates were adjusted for study site, age group, sex, race/ethnicity, self-rated general health, and number of days from illness onset to enrollment using logistic regression. Interim VE estimates for the 2016–17 season were based on patients enrolled through February 4, 2017.

Among the 3,144 children and adults with ARI enrolled at the five study sites from November 28, 2016, through February 4, 2017, 744 (24%) tested positive for influenza virus by rRT-PCR; 656 (88%) of these viruses were influenza A, and 90 (12%) were influenza B viruses (Table 1). Among 606 subtyped influenza A viruses, 595 (98%) were A (H3N2) viruses. The proportion of patients with influenza differed by study site, sex, age group, race/ethnicity, and interval from illness onset to enrollment (Table 1). The proportion vaccinated ranged from 46% to 61% across sites and differed by sex, age group, and interval from illness onset to enrollment.

**TABLE 1:** Selected characteristics for enrolled patients with medically attended acute respiratory illness, by influenza test result and seasonal influenza vaccination status — U.S. Influenza Vaccine Effectiveness Network, United States, November 28, 2016–February 4, 2017

Characteristic	Influenza test result		p-value^†^	Vaccination status		p-value^†^
	No. positive (%)	No. negative (%)		No. enrolled	No. vaccinated*(%)	
Overall	744 (24)	2,400 (76)		3,144	1,650 (52)	
**State of study site**						
Michigan	92 (26)	267 (74)	<0.001	**359**	206 (57)	<0.001
Pennsylvania	176 (30)	416 (70)		**592**	271 (46)	
Texas	56 (8)	646 (92)		**702**	341 (49)	
Washington	374 (38)	613 (62)		**987**	598 (61)	
Wisconsin	46 (9)	458 (91)		**504**	234 (46)	
**Sex**						
Male	350 (26)	990 (74)	0.005	**1,340**	665 (50)	0.006
Female	394 (22)	1410 (78)		**1,804**	985 (55)	
**Age group**						
6 mos–8 yrs	97 (14)	614 (86)	<0.001	**711**	362 (51)	<0.001
9–17 yrs	122 (33)	247 (67)		**369**	128 (35)	
18–49 yrs	208 (21)	783 (79)		**991**	452 (46)	
50–64 yrs	189 (31)	425 (69)		**614**	337 (55)	
≥65 yrs	128 (28)	331 (72)		**459**	371 (81)	
**Race/Ethnicity** ^§^						
White	532 (23)	1,744 (77)	<0.001	**2,276**	1,231 (54)	0.001
Black	81 (35)	153 (65)		**234**	99 (42)	
Other race	72 (24)	222 (76)		**294**	163 (55)	
Hispanic	47 (15)	274 (85)		**321**	150 (47)	
**Self-rated health status**						
Fair or poor	55 (22)	200 (78)	0.52	**255**	142 (56)	0.04
Good	179 (23)	599 (77)		**778**	436 (56)	
Very good	301 (25)	902 (75)		**1,203**	622 (52)	
Excellent	209 (23)	699 (77)		**908**	450 (50)	
**Illness onset to enrollment (days)**						
<3	284 (29)	693 (71)	<0.001	**977**	473 (48)	0.003
3–4	304 (25)	933 (75)		**1,237**	654 (53)	
5–7	156 (17)	774 (83)		**930**	523 (56)	
**Influenza test result**						
Negative	—	2,400	—	**2,400**	1,317 (55)	—
Influenza B positive^¶^	90	—		**90**	23 (26)	
B/Yamagata	83	—		**83**	20 (24)	
B/Victoria	4	—		**4**	1 (25)	
B lineage pending	3	—		**3**	2 (67)	
Influenza A positive^¶^	656	—		**656**	310 (47)	
A (H1N1)pdm09	11	—		**11**	3 (27)	
A (H3N2)	595	—		**595**	282 (47)	
A subtype pending	50	—		**50**	25 (50)	

* Defined as having received ≥1 dose of influenza vaccine ≥14 days before illness onset. A total of 89 participants who received the vaccine ≤13 days before illness onset were excluded from the study sample.

^†^ The chi-square statistic was used to assess differences between the numbers of persons with influenza-negative and influenza-positive test results, in the distribution of enrolled patient and illness characteristics, and in differences between groups in the percentage vaccinated.

^§^ Enrollees were categorized into one of four mutually exclusive racial/ethnic populations: white, black, other race, and Hispanic. Persons identified as Hispanic might have been of any race. Persons identified as white, black, or other race were non-Hispanic. Race/ethnicity data were missing for 19 enrollees.

^¶^ Two patients had coinfection with influenza A and influenza B, making the sum 746, or two greater than the total number of influenza positives.

The proportion of ARI patients vaccinated with 2016–17 seasonal influenza vaccine was 45% among influenza patients compared with 55% among influenza-negative participants (Table 2). After adjusting for study site, age group, sex, race/ethnicity, self-rated general health, and number of days from illness onset to enrollment, VE against medically attended ARI because of influenza was 48% (CI = 37%–57%). VE for all ages was 43% (CI = 29%–54%) against medically attended ARI because of A (H3N2) virus infection and 73% (CI = 54%–84%) against influenza B virus infection. VE point estimates against H3N2-related illness varied by age group; statistically significant protection was found against H3N2-related illness among children aged 6 months through 8 years (VE = 53%; CI = 16%–74%) and adults aged 50–64 years (VE = 50%; CI = 23%–67%), whereas protection in other age groups did not reach statistical significance.

**TABLE 2:** Number and percentage receiving 2016–17 seasonal influenza vaccine among 3,144 outpatients with acute respiratory illness and cough, by influenza test result status, age group, and vaccine effectiveness against all influenza A and B and against virus types A (H3N2) and B — U.S. Influenza Vaccine Effectiveness Network, United States, November 28, 2016–February 4, 2017

Influenza type/Age group	Influenza-positive		Influenza-negative		Vaccine effectiveness*	
	Total	No. (%) vaccinated	Total	No. (%) vaccinated	Unadjusted % (95% CI)	Adjusted % (95% CI)
**Influenza A and B**						
**Overall**	744	333 (45)	2,400	1,317 (55)	33 (21 to 44)^†^	48 (37 to 57)^†^
**Age group**						
6 mos–8 yrs	97	32 (33)	614	330 (54)	58 (33 to 73)^†^	53 (22 to 72)^†^
9–17 yrs	122	36 (30)	247	92 (37)	29 (−12 to 56)	32 (−20 to 61)
18–49 yrs	208	89 (43)	783	363 (46)	13 (−18 to 36)	19 (−17 to 43)
50–64 yrs	189	76 (40)	425	261 (61)	58 (40 to 70)^†^	58 (38 to 72)^†^
≥65 yrs	128	100 (78)	331	271 (82)	21 (−31 to 52)	46 (4 to 70)^†^
**Influenza A (H3N2)**						
**Overall**	595	282 (47)	2,400	1,317 (55)	26 (11 to 38)^†^	43 (29 to 54)^†^
**Age group**						
6 mos–8 yrs	68	24 (35)	614	330 (54)	53 (21 to 72)^†^	53 (16 to 74)^†^
9–17 yrs	94	28 (30)	247	92 (37)	29 (−19 to 57)	23 (−43 to 59)
18–49 yrs	168	73 (43)	783	363 (46)	11 (−24 to 36)	13 (−30 to 41)
50–64 yrs	154	70 (45)	425	261 (61)	48 (24 to 64)^†^	50 (23 to 67)^†^
≥65 yrs	111	87 (78)	331	271 (82)	20 (−37 to 53)	44 (−3 to 69)
**Influenza B**						
**Overall**	90	23 (26)	2,400	1,317 (55)	72 (54 to 83)^†^	73 (54 to 84)^†^

**Abbreviation:** CI = confidence interval.

* Vaccine effectiveness was estimated as 100% x (1 - odds ratio [ratio of odds of being vaccinated among outpatients with influenza-positive test results to the odds of being vaccinated among outpatients with influenza-negative test results]); odds ratios were estimated using logistic regression.

^†^ Statistically significant at the p<0.05 level.

As of February 10, 2017, a total of 13 influenza A (H3N2) viruses from U.S. Flu VE Network participants had been characterized by CDC; 11 (85%) belonged to genetic group 3C.2a or the related group 3C.2a1, and all of those characterized antigenically were similar to the reference virus representing the 2016–17 A (H3N2) vaccine component.

## Discussion

Interim influenza vaccine effectiveness estimates for the 2016–17 season indicate that vaccination reduced the risk for influenza-associated medical visits by approximately half. Influenza activity is likely to continue for several more weeks in the United States, and vaccination efforts should continue as long as influenza viruses are circulating. Persons aged ≥6 months who have not yet received the 2016–17 influenza vaccine should be vaccinated as soon as possible.^§^ As of February 3, 2017, approximately 145 million doses of influenza vaccine had been distributed in the United States for the 2016–17 season.

Interim VE estimates indicate improved protection during the 2016–17 influenza season against the predominant influenza A (H3N2) virus belonging to genetic group 3C.2a, which emerged in early 2014 and was predominant during the 2014–15 influenza season in the United States. During 2014–15, these influenza A (H3N2) 3C.2a viruses were antigenically different from the recommended A (H3N2) vaccine component, and this resulted in low (1%) vaccine effectiveness against illness caused by influenza A (H3N2) 3C.2a viruses (*4*). Low effectiveness of the 2014–15 vaccines likely contributed to high rates of influenza-associated hospitalizations that season, especially among adults aged ≥65 years. In contrast, rates of influenza-associated hospitalizations observed to date have been substantially lower during the 2016–17 season (*2*). Virologic surveillance indicates that the majority of influenza A (H3N2) viruses collected by U.S. laboratories during the 2016–17 season remain antigenically similar to the A/Hong Kong/4801/2014–like cell propagated reference virus belonging to genetic group 3C.2a, which is the recommended influenza A (H3N2) component of the 2016–17 Northern Hemisphere vaccine. 

Since the 2009 influenza A (H1N1) pandemic, VE estimates for A (H3N2) viruses have been lower than VE estimates against A (H1N1) and influenza B viruses. Interim VE estimates against illness caused by influenza A (H3N2) viruses during the 2016–17 influenza season are similar to U.S. VE estimates against A (H3N2)-related illness during the 2011–12 and 2012–13 seasons (VE = 39%) (*5*,*6*). Also, a meta-analysis of VE studies using the test-negative design conducted from the 2007–08 through the 2014–15 influenza seasons reported a pooled VE estimate against A (H3N2)-related illness of 33% (CI = 26%–39%), compared with 61% (CI = 57%–65%) against influenza A (H1N1)pdm09 and 54% (CI = 46%–61%) against influenza B virus–related illness (*7*). These results reflect properties unique to A (H3N2) viruses that pose special challenges. Influenza A (H3N2) viruses undergo more frequent and extensive genetic changes than do influenza A (H1N1) and influenza B viruses, and require more frequent updates to the A (H3N2) vaccine virus components to maintain activity against evolving circulating strains. In addition, A (H3N2) viruses continue to undergo changes in their receptor-binding specificity, which might result in genetic changes during growth in eggs. Most influenza vaccines are manufactured using egg-based production processes. These genetic changes (referred to as egg-adapted changes) alter the antigenic properties of candidate vaccine viruses (CVVs) as they are grown in eggs and potentially during the vaccine production process (*8*). The egg-adapted changes might contribute to the lower vaccine effectiveness seen with A (H3N2) viruses compared with A (H1N1) and B viruses. Efforts are ongoing to improve influenza vaccine effectiveness against A (H3N2) viruses in CVV development and in manufacturing.

As of February 10, 2017, influenza activity remained elevated nationally and was widespread across most of the United States. During recent A (H3N2) virus predominant–seasons, persons aged ≥65 years and young children experienced higher rates of severe illness and influenza-associated hospitalization compared with other age groups. With vaccine effectiveness of 48%, some vaccinated persons will become infected with influenza. Clinicians should maintain a high index of suspicion for influenza infection among persons with acute respiratory illness while influenza activity is ongoing, especially among older adults. Early antiviral treatment can reduce severity and complications of influenza-associated illness (*9*). Early antiviral treatment is recommended for persons with suspected influenza with severe or progressive illness (e.g., hospitalized persons) and persons at high risk for complications from influenza, such as children aged <2 years, adults aged ≥65 years and persons with underlying health conditions,^¶^ even if illness is less severe. Antiviral medications should be used as recommended for treatment in patients with suspected influenza, regardless of vaccination status. The decision to initiate antiviral treatment should not be delayed while waiting for laboratory confirmation of influenza and should not be dependent on insensitive assays, such as rapid influenza diagnostic tests.

The findings in this report are subject to at least four limitations. First, vaccination status included self-report at four of five sites. End-of-season VE estimates based on updated documentation of vaccination status might differ from interim estimates. Second, information from medical records and immunization registries is needed to evaluate VE by vaccine type and for fully vaccinated compared with partially vaccinated children (children aged <9 years require 2 vaccine doses during their first vaccination season), as well as to evaluate the effects of prior season vaccination and timing of vaccination; end-of-season analysis of VE by vaccine type and effects of partial or prior season vaccination is planned. Third, an observational study design has greater potential for confounding and bias relative to randomized clinical trials. However, the test-negative design is widely used in VE studies and has been used by the U.S. Flu VE Network to estimate VE for the past several influenza seasons. Finally, small sample sizes in some age groups resulted in wide confidence intervals, and end-of-season VE estimates could change as additional patient data become available or if there is a change in circulating viruses late in the season. It is also important to note that the VE estimates in this report are limited to the prevention of outpatient medical visits, rather than more severe illness outcomes, such as hospitalization or death; data from studies measuring VE against more severe outcomes will be available at a later date.

Annual vaccination against circulating influenza viruses remains the best strategy for preventing illness from influenza. As of early November 2016, only 37% of children aged 6 months–17 years, 37% of adults aged 18–64 years, and 57% of adults aged ≥65 years had received influenza vaccine this season (*10*). Among pregnant women, early estimates for 2016–17 indicated that only 47% had been vaccinated by early November 2016 (*10*). In addition to ongoing vaccination efforts, antiviral medications continue to be an important adjunct to the treatment and control of influenza and should be used as recommended, regardless of patient vaccination status.

## Footnotes

* The U.S. Flu VE Network sites and the date enrollment began are as follows: Group Health Cooperative (Seattle, Washington) (November 28, 2016); Marshfield Clinic Research Foundation (Marshfield, Wisconsin) (January 3, 2017); University of Michigan School of Public Health (the School of Public Health partnered with the University of Michigan Health System, Ann Arbor, and the Henry Ford Health System, Detroit, Michigan) (January 3, 2017); University of Pittsburgh Schools of the Health Sciences (the Schools of the Health Sciences partnered with the University of Pittsburgh Medical Center, Pittsburgh, Pennsylvania) (December 5, 2016); and Baylor Scott and White Health, Texas A&M University Health Sciences Center College of Medicine (Temple, Texas) (December 13, 2016).

† 100% x (1 - odds ratio [ratio of odds of being vaccinated among outpatients with influenza-positive test results to the odds of being vaccinated among outpatients with influenza-negative test results]).

§ A local influenza vaccine provider can be found by accessing the Flu Vaccine Finder website at https://vaccinefinder.org/?address.

¶ A complete summary of guidance for antiviral use is available at https://www.cdc.gov/flu/professionals/antivirals/summary-clinicians.htm. Groups at high risk for influenza complications include the following: children aged <2 years; adults aged ≥65 years; persons with chronic pulmonary conditions (including asthma); cardiovascular disease (except hypertension alone); persons with renal, hepatic, or hematologic (including sickle cell) disease; persons with metabolic disorders (including diabetes mellitus); persons with neurologic and neurodevelopmental conditions (including disorders of the brain, spinal cord, peripheral nerves and muscles, such as cerebral palsy, epilepsy [seizure disorders], stroke, intellectual disability [mental retardation], moderate to severe developmental delay, muscular dystrophy, or spinal cord injury); persons with immunosuppression, including that caused by medications or by human immunodeficiency virus infection; women who are pregnant or postpartum (within 2 weeks after delivery); persons aged <19 years who are receiving long-term aspirin therapy; American Indian/Alaska Natives; persons with morbid obesity (i.e., body-mass index ≥40); and residents of nursing homes and other chronic-care facilities**.**
